# Variations in respiratory and functional symptoms at four months after hospitalisation due to COVID-19: a cross-sectional study

**DOI:** 10.1186/s12890-024-02866-5

**Published:** 2024-01-31

**Authors:** Monika Fagevik Olsén, Louise Lannefors, Ewa-Lena Johansson, Hanna C. Persson

**Affiliations:** 1 Department of Health and Rehabilitation/Physiotherapy, Institute of Neuroscience and Physiology, Box 455, Sahlgrenska Academy, University of Gothenburg, 405 30 Gothenburg, SE Sweden; 2 Department of Physiotherapy, Sahlgrenska University Hospital, 413 45 Gothenburg, SE Sweden; 3 Department of Clinical Neuroscience, Institute of Neuroscience and Physiology, Sahlgrenska Academy, University of Gothenburg, Gothenburg, Sweden

**Keywords:** Breathing pattern, Dysfunctional breathing, Long-COVID, Physiotherapy evaluation, Post-COVID condition, Respiratory symptoms

## Abstract

**Background:**

Much remains unknown about complex respiratory symptoms after COVID-19. Here we aimed to describe and analyse patients’ various respiratory symptoms 4 months after discharge from hospitalisation for COVID-19, focusing on sex, previous pulmonary disease, and prolonged mechanical ventilation.

**Methods:**

This cross-sectional study involved five hospitals and included 52 patients with self-assessed respiratory dysfunction at 4 months after discharge from hospitalisation for severe COVID-19. Their average age was 63 years, 38% were women, 15 had a previous diagnosed pulmonary disease, and 29 were current or previous smokers. Additionally, 31 had required intensive care—among whom 21 were intubated and 11 needed mechanical ventilation for ≥20 days. Respiratory function was tested concerning lung volumes, expiratory flow, muscle strength, physical capacity (including concurrent oxygen saturation), thoracic expansion, and respiratory movements.

**Results:**

Among 52 patients, 47 (90%) had one or several objectively measured respiratory function abnormalities. Decreased thoracic expansion was observed in 32 patients (62%), abnormal respiratory movements in 30 (58%), decreased vital capacity in 21 (40%), low physical function in 13 (26%), and desaturation during the test in 9 (17%). Respiratory inspiratory muscle strength was more commonly diminished than expiratory strength (27% vs. 8%). We did not observe differences between men and women, or between patients with versus without diagnosed pulmonary disease, except that those with pulmonary disease had significantly lower physical capacity assessed with 6MWD (70% vs. 88% predicted, *p* = 0.013). Compared to those who did not, patients who required ≥20 days of mechanical ventilation performed similarly on most tests, except that all thoracic breathing movements were significantly smaller (*p* < 0.05). The numbers and combinations of abnormal findings varied widely, without clear patterns.

**Conclusion:**

Patients with remaining respiratory symptoms 4 months after discharge from hospitalization due to COVID-19 may suffer from various abnormal breathing functions, and dysfunctional breathing that is not detected using traditional measurements. These patients may benefit from multidimensional measuring of breathing movement, thoracic expansion, and respiratory muscle strength, along with traditional measurements, to assess their symptoms and enable prescription of optimal treatment interventions and rehabilitation.

**Trial registration:**

FoU i Sverige (Research & Development in Sweden, Registration number: 274476, registered 2020-05-28).

## Introduction

As people continue to experience COVID-19, the number of patients reporting persisting symptoms becomes an increasing burden to public health [1–3]. The epidemiologic explanations and predictors of lingering COVID-19 effects remain unknown [1, 4–6]. In the long-term condition, patients report one or more of a variety of new, recurrent, or ongoing symptoms, which may vary over time [7–10]. In most long-term studies, the prevalence of remaining symptoms is higher among women [4, 5, 8, 11]. The most common respiratory symptoms include dyspnoea, shortness of breath or difficulties in breathing, persistent cough, and reduced exercise tolerance [1, 2, 5, 7, 8]. Symptoms may affect a person’s ability to perform daily activities and make it difficult to function during everyday life [8, 12, 13].

Scientific studies are increasingly accepting self-reported respiratory symptoms among patients after discharge from hospitalisation due to COVID-19 [2, 3, 9, 14]. Notably, in most investigations, the laboratory and imaging tests of respiratory symptoms have revealed no abnormalities or have been non-diagnostic [1, 7, 8, 12, 15].

Among people who are not hospitalised due to COVID-19, we have previously demonstrated that specific measurements may be necessary in addition to traditional tests, when aiming to detect remaining or reappearing respiratory symptoms [16]. An expanded test battery for non-hospitalised patients included measurements of respiratory movement, breathing pattern, thoracic expansion, and respiratory muscle strength. The results revealed decreased strength of both inspiratory and expiratory muscles (64 and 17% of predicted, respectively) and an abnormal breathing pattern in 57 of the 60 patients (95%). It remains unknown whether this pattern is also present in patients who were hospitalised due to more severe primary consequences of COVID-19.

In the present study, we aimed to describe and analyse patients’ various respiratory symptoms and signs at 4 months after discharge from hospitalisation due to COVID-19. We particularly focused on the potential impact of sex, previous diagnosed pulmonary disease, and prolonged mechanical ventilation.

## Method

### Study design, and participants

This cross-sectional study included patients with remaining self-assessed respiratory dysfunction 4 months after discharge from hospitalisation due to severe COVID-19. The subjects were enrolled in the study *Life in the time of Covid-study in Gothenburg, Sweden* (GOT-LOCO) [17], which had the following inclusion criteria: hospitalised due to COVID-19 within the Västra Götaland Region (VGR), non-contagious at inclusion, expected hospital stay of > 5 days, age ≥ 18 years, and independent living prior to hospitalisation. The exclusion criteria were inability to give informed consent, severe illness of other kind with expected high 1-year mortality, and not being a Swedish resident.

**Fig. 1:**
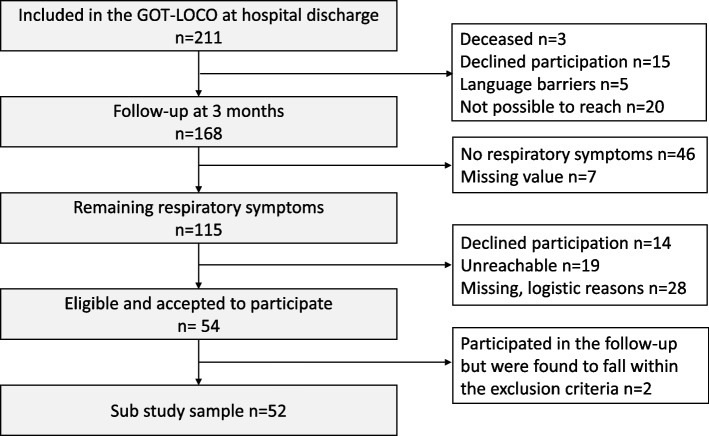
Flowchart

A total of 211 participants were included in the GOT-LOCO study during the recruitment period of July 9, 2020, to February 23, 2021 (i.e. during the first and second waves of the COVID-19 pandemic). Participants received hospital care at one of the five hospitals in the region (VGR: 1.67 million inhabitants). Within the GOT-LOCO study, all participants were intended to be contacted by telephone approximately 3 months after hospital discharge, of which 168 were successfully reached (Fig. 1).

Participants were asked questions regarding their current health situation, and the main results have been published elsewhere [17]. All the 168 participants were assessed with the COPD Assessment Test (CAT) [18] slightly modified to suit patients with COVID-19 [16]. Participants (*n* = 115) with total CAT score ≥ 10, or reported perceived problems (≥3, indicating primary moderate – severe symptoms) in following questions: cough, mucus, chest tightness, breathlessness during activity and limitations doing activities at home (CAT item 1–5) were offered to be contacted for an appointment and a follow-up by a physiotherapist.

Patients who agreed to participate in the follow-up including a physical examination were contacted by phone by the physiotherapist. Some patients could not be reached by phone, although repeated attempts were made. Finally, 54 patients agreed to participate. The physical examination was performed at Sahlgrenska University Hospital, Gothenburg, by one of the two involved certified physiotherapists specialised in respiration. A standardised protocol was followed, including assessments of lung function, respiratory muscle strength, breathing pattern, and physical condition. Additionally, the participants answered questionnaires concerning respiratory symptoms and functional limitations. During the study time period, the participants were asked to maintain their use of any drugs they might have been prescribed prior to this follow-up. To enable evaluation of remaining symptoms after COVID-19, another exclusion criterion was added after the follow-up: severe pre-infectious comorbidity affecting breathing function. This criterion led to the exclusion of two patients who participated in the follow-up, leaving 52 patients included in the analyses.

The following sections describe the components of the standardised physical examination.

### Lung function assessed with spirometry

Each subject performed a spirometry test using a portable ultra-sonic spirometer (Easy One ndd, Medical Technologies, Switzerland). The obtained measurements included forced vital capacity (FVC), forced expiratory volume in 1 second (FEV_1_), forced inspiratory vital capacity (FIVC), and peak expiratory flow (PEF). Tests were performed with patients in a seated position, feet on the floor, and with a nose clip, following the recommendations of the European Respiratory Society [19, 20]. The results were analysed as the percentage of predicted reference values based on gender, age, and height, in accordance with the reference material for spirometry [21].

### Respiratory muscle strength

The maximal inspiratory pressure (MIP) and the maximal expiratory pressure (MEP) were measured in a standardised manner using a micro-RPM (Care Fusion, Yorba Linda, CA, USA), and expressed as cm H_2_O. While in a seated position, feet on the floor, and with a nose clip, subjects were asked to perform a maximal inspiration manoeuvre from residual volume to measure MIP, and a maximal expiration manoeuvre from total lung capacity to measure MEP. At least three manoeuvres were performed for each test, to ensure that the difference between the two best values was < 10% [22]. Reference values and the lower limit of normal were defined for MIP according to Sclauser Pessoa et al. [23], and for MEP according to Bissett et al. [24].

### The 6-minute walk distance

The 6-minute walk distance (6MWD) was performed walking along a 30-m-long indoor track back and forth in a standardised manner [25]. The lower limit of normal, and predicted distance on the 6MWD, were determined according to Enright et al. [26].

### The 1-minute sit-to-stand test (1MSTS)

From a chair with a height of 45 cm, subjects were asked to perform as many sit-to-stand manoeuvres as possible in 1 minute, in a standardised manner, with feet on the floor, and arms crossed over the chest. The number of stand-ups was reported, and the predicted values were determined according to Strassman et al. [27].

Before, during, and after the test, peripheral oxygen saturation (SpO_2_) was recorded using a Rad-57 oximeter (Masimo Corporation, Irvine CA, USA). A level of < 92% was defined as desaturation, in accordance with clinical practice at the hospital.

### Thoracic expansion

Using a measuring tape (marked in mm), thoracic expansion was assessed as the circumference at the level of Proc Xiphoideus, with the patient in a standing position with hands on the head. Participants were instructed to “Breathe in maximally and make yourself as big as possible” and “Breathe out maximally and make yourself as small as possible”. Since no lower limit of normal was found, a value below 80% of predicted was defined as decreased [28, 29].

### Respiratory movement

Respiratory movements were recorded in a supine position, using the Respiratory Movement-Measuring Instrument (RMMI; ReMo, Reykjavik, Iceland) [30]. First, during 30 seconds of tidal volume breathing, and then during 60 seconds of performing maximal breathing manoeuvres interspaced by tidal volume breathing. The RMMI records bilateral changes in the anterior posterior diameter, including both upper and lower thoracic and abdominal movements. The subjects were not aware of when data were recorded during tidal volume breathing. During the deep-breathing manoeuvres, participants were instructed to perform cycles of maximal breaths, accompanied by resting tidal volume breathing. Reference values were based on the study by Ragnarsdottir et al. [30].

### Degree of functional limitation

To assess the patients’ subjective experience of their degree of functional limitation, we used the Swedish version of The Post-Covid Functional Status (PCFS) [31].

### Data analysis or statistical methods

Data management and analysis were performed using SPSS Statistics 25 (IBM).

Descriptive statistics were presented as mean, standard deviation, median, minimum, maximum, number, and percentage. Group comparisons were analysed using Student’s t-test and the Mann-Whitney U-test or Chi^2^ test for continuous and categorical variables, respectively. If the data were not normally distributed, non-parametric tests were used. The significance level was set to *p* < 0.05. Spearman’s correlation coefficient was calculated to assess the correlation between thoracic expansion and spirometry. Correlation was defined as: none to little (r_s_ < 0.025); low (r_s_ of 0.26–0.49); moderate (r_s_ of 0.50–0.69); high (r_s_ of 0.70–0.89); or very high (r_s_ of 0.9–1.00) [32].

## Results

A total of 115 patients had remaining or re-appearing respiratory symptoms at 4 months after discharge from the hospital. Of these patients, 52 (45%) were included in the present analysis (Fig. 1). The participants (*n* = 52) and non-participants did not significantly differ in age (*p* = 0.061), sex (*p* = 0.429), or number of days hospitalised due to COVID-19 (*p* = 0.361). Participants had a median age of 63.5 years, and 20 (38%) were female (Table 1). Among the 52 participants, 15 (29%) had been diagnosed with pulmonary disease before the infection. Concerning smoking history, 4 were current and 25 former smokers, such that 56% had been exposed to smoking. The mean BMI was 29.5 kg/m^2^ for the whole group, of whom 18 (35%) were obese (BMI > 30 kg/m^2^). During hospitalisation, 31 had required intensive care management, of whom 21 were intubated and 11 required mechanical ventilation for ≥20 days (hereafter defined as prolonged mechanical ventilation). Men and women did not significantly differ in the length of hospital stay (*p* = 0.35) or need for ICU admission (*p* = 0.38). However, men more commonly required prolonged mechanical ventilation (*p* = 0.029) and tracheostomy (*p* = 0.026) (Table 1).

**Table 1:** Descriptive characteristics of the study sample. Mean (SD) and median (min-max) or n (%)

	Total sample *n* = 52	Men *n* = 32	Women *n* = 20	*p*-value
**Age at infection,** *years*				
Mean (SD)	62.9 (11.0)	62.3 (8.3)	63.7 (14.6)	0.579
Median (min-max)	63.5 (31–93)	63.5 (43–77)	64.0 (31–93)	
**BMI**				
Mean (SD)	29.5 (6.2)	30.1 (5.2)	28.6 (7.7)	0.096
Median (min-max)	28.6	29.0	26.1	
	(18.7–51.4)	(19.0–44.8)	(18.7–51.4)	
**Length of stay,** *days*				
Median (min-max)	22 (5–175)	23 (5–175)	22.0 (6–90)	0.351
**Need of ICU care**				
Yes (%)	31 (60)	21 (66)	10 (50)	0.384
No (%)	21 (40)	11 (34)	10 (50)	
**Intubated**				
Yes (%)	21 (40)	15 (47)	6 (30)	0.247
No (%)	31 (60)	17 (53)	14 (70)	
**If yes, intubated, days (%)**				
1–5	3 (14)	2 (13)	1 (17)	0.029
6–10	4 (20)	1 (6)	3(49)	
11–15	1 (5)	0	1 (17)	
16–20	3 (14)	2 (13)	1 (17	
21–30	3 (14)	3 (20)	0	
> 31	6 (28)	6 (40)	0	
Unclear	1 (5)	1 (6)	0	
**HFNC/Optiflow, days (%)**				
Yes	35 (67)	23 (72)	12 (60)	0.953
No	14 (27)	7 (22)	7 (35)	
Unclear	3 (6)	2 (6)	1 (5)	
**Tracheostomy, days (%)**				
Yes	14 (27)	12 (38)	2 (10)	0.026
No	38 (73)	20 (62)	18 (90)	
**Pulmonary disease’**				
In total	15	8	7	0.534
COPD	7	2	2	
Asthma	8	5	3	
Sarcoidosis	1	1	0	
**Smoker**				
Yes (%)	4	1	3	0.235
No (%)	23	16	7	
Former smoker (%)	25	5	9	
**Physical activity prior Covid-19 infection (SGPALS)**				
* Physically inactive,* n (%)	17 (33)	10 (31)	7 (35)	0.594
* Light physical activity,* n (%)	22 (42)	13 (41)	9 (45)	
* Moderate physical activity,* n (%)	13 (25)	9 (28)	4 (20)	
* Vigorous physical activity,* n (%)	0 (0)	0 (0)	0 (0)	
**3 months post discharge**				
**Phone follow-up, days median (min-max) since discharge**	94 (74–131)	92.5 (76–119)	95 (74–131)	
**CAT**				
Total score median, (mi-max)	18 (6–39)	32 (6–39)	22 (8–32)	0.199
* Cough*	2 (0–5)	2 (0–5)	2 (0–4)	
* Mucus*	2 (0–5)	2 (0–5)	1 (0–4)	
* Chest tightness*	2 (0–5)	2 (0–5)	3 (0–5)	
* Dyspnoea during activity*	4 (1–5)	4 (1–5)	4 (2–5)	
* Limitations at home*	3 (0–5)	2 (0–5)	3 (0–5)	
**Physical activity (SGPALS)**				
* Physically inactive,* n (%)	17 (33)	8 (25)	9 (45)	0.153
* Light physical activity,* n (%)	23 (44)	15 (47)	8 (40)	
* Moderate physical activity,* n (%)	10 (19)	7 (22)	3 (15)	
* Vigorous physical activity,* n (%)	2 (4)	2 (6)	0 (0)	
**PCFS**				
Severe limitations	2	1	1	0.075
Moderate limitations	7	3	4	
Slight limitations	14	7	7	
Negligible limitation	14	18	4	
No limitations	22 6	3	3	

*BMI* Body Mass Index: *ICU* Intensive care unit: *CAT* Chronic Obstructive Pulmonary Disease test: *SGPASL* Saltin-Grimby Physical Activity Level Scale 1: PCFS: Post Covid Functional Status

Combinations possible

The degree of functional limitation, as determined by the PCFS scale, was reported as “none or negligible” in 28 patients (54%), “slight” in 14 patients (27%), “moderate” in 7 patients (14%) and “severe” in 2 patients (4%). The median score of the modified CAT scale was 20 (range 3–31). A feeling of tightness (median score of 2) around the chest was reported by 21 subjects (40%) (Table 1).

Table 2 displays a comparison of the measured respiratory function data, % predicted of 6MWD and 1MSTS, and SpO_2_ changes during the 1MSTS at 4 months after hospital discharge—with stratification according to gender, patients with and without previous pulmonary disease, and patients who did or did not require mechanical ventilation for ≥20 days. Overall, the data showed that the measured FVC was below the lower limit of normal (LLN) in 21 patients (40%), FEV1 was reduced in 15 patients (29%), and FIVC was reduced in 6 patients (12%). Lung volumes (% predicted) did not significantly differ according to sex, presence of pulmonary disease before COVID-19, or duration of mechanical ventilation.

**Table 2:** Comparison of measured respiratory function data, 6MWD and 1MSTS, as well as SpO2 changes during 1MSTS related to gender, with and without previous pulmonary disease, and those patients who required mechanical ventilation for > or < than 20 days, at 4 months post-hospital discharge. Mean (SD) of % predicted, median [min-max]

Variable		All *n* = 52	Sex			Previous pulmonary disease			Mechanical ventilation		
			Men *n* = 32	Women *n* = 20	*p*-value	Yes *n* = 15	No *n* = 37	*p*-value	< 20 days *n* = 41	≥20 days *n* = 11	*p*-value
FVC	% pred	77.2 (16.6)*** 79.0 [32–114]	75.2 (15.4)*** 76.2 [34–106]	80.5 (18.4)*** 81.0 [32–114]	0.153	78.8 (14.7) 77.0 [46–100]	77.8 (17.5) 79.5 [32–114]	0.512	78.0 (16.0) 79.4 [32–114]	74.3 (19.2) 78.5 [34–106]	0.499
	<LLN, n (%)	21 (40.5%)	14 (43.8%)	7 (35.0%)	0.575	8 (53.3%)	13 (35.1%)	0.350	16 (39.0%)	5 (45.5%)	0.739
FEV1	% pred	79.5 (20.7)*** 79.3 [23–121]	78.3 (18.7)*** 78.7 [33–115]	81.4 (23.9)** 85.6 [23–121]	0.301	73.8 (22.6) 78.5 [32–103]	81.8 (19.7) 81–5 [23–121]	0.499	79.4 (19.8) 79.0 [23–121]	79.8 (24.8 [88.7 [33–115]	0.327
	<LLN, n (%)	15 (28.8%)	11 (34.4%)	4 (20.0%)	0.352	6 (40%)	9 (24%)	0.318	11 (26.8%)	4 (36.3%)	0.709
FIVC	% pred	102.6 (23.4) 106.3 [27–146]	99.4.1 (22.0) 102.1 [49–146]	107.7 (25.4)** 113.4 [27–146]	**0.080**	104.4 (20.0) 102.6 [77–143]	101.9 (24.8) 106.9 [27–146]	0.589	102.9 (23–1) 107.54 [27–143]	101.6 (25.5) 102.3 [49–146]	0.966
	<LLN, n (%)	6 (11.5%)	5 (16.1%)	1 (5.3)	0.387	1 (6.7%)	5 (13.5%)	0.663	5 (12.8%)	1 (9.1%)	0.737
MIP	% pred	90.3 (29.3)** 91.2 [16–192]	97.1 (25.3) 96.9 [43–192]	79.3 (32.4)* 87.5 [16–149]	0.060	87.5 (31.1) 89.2 [23–149]	91.4 (28.9) 91.9 [16–192]	0.607	86.7 (30.7) 89.3 [16–192]	103.7 (18.7) 102.8 [79–149]	0.607
	<LLN, n (%)	14 (26.9)	6 (18.8)	8 (40.0)	0.116	6 (40%)	8 (21.6%)	0.190	14 (34.1)	0	**0.025**
MEP	% pred	99.5 (29.6) 99.7 [9–163]	94.8 (20.0) 94.1 (56–143)	107.4 (40.2) 113 (9–163)	0.112	98.2 (32.4) 94.5 (57–161]	100.1 (28.9) 102.1 [9–163]	0.517	97.6 (31.3) 96.0 [9–163]	106.4 (22.6) 102.1 [81–161]	0.517
	<LLN, n (%)	4 (7.8)	0	4 (20.0)	0.017	2 (13.3%)	2 (5.4%)	0.310	4 (10.3%)	0	0.563
6MWD§	% pred	82.4 (23.6) 85.1 [11–177]	82.4 (15.5) 81.8 [57–126]	82.3 (34.0) 85.7 [11–177]	0.868	70.0 (24.8) 73.5 [11–107]	87.9 (21.2) 87.2 [57–177]	**0.028**	83.8 (25.2) 87.2 [11–177]	77.1 (16.0) 70.65 [63–107]	**0.028**
	<LLN,n (%)	13 (26.5)	9 (29.0)	4 (22.2)	0.743	7 (46.7%)	6 (17.6%)	0.081	9 (23.1%)	4 (40%)	0.422
1MSTS	% pred	68.2 (24.7) 66.7 [13–151]	71.3 (24.7) 66.7 [29–151]	63.3 (24.7) 67.6 [13–104]	0.288	62.8 (31.3) 56.8 [29–151]	70.3 (21.9) 67.6 [13–112]	0.125	70.6 (26.8) 70.6 [13–151]	59.6 (12.1) 61.4 [41–77]	0.125
	<LLN, n (%)	35 (72.9)	22 (73.3)	13 (72.2)	0.933	12 (92.3%)	23 (66.7%)	0.081	24 (64.8%)	11 (100%)	**0.023**
SaO2^a^	Difference during 1 min STS-test, %	−2.2 (2.7) −2 [−13–2]	−2,5 (2.98) −2 [−13–2]	−1.8 (2.1) −1 [−5–1]	0.476	−2.5 (2.7) − 2 [−8–1}	−1.8 (2.0) − 1.5 [−5–2]	0.409	−1.95 (2.2) − 1.0 [−8–2)	− 2.1 (2.2) − 2.0 [− 5–0]	0.409
	SaO2 < 92%, n (%)	9 (18)	7 (22.5)	2 (10.5)	0.452	5 (33.3%)	3 (16.7%)	**0.039**	6 (14.6%)	2 (20%)	0.647
Thoracic expansion	% pred	63.3 (26.8)*** 58.3 [10–150]	62.3 (24.9)*** 51.7 [33–150]	64.7 (30.2)** 60 [10–120]	0.706	58.9 (23.6) 53.3 [10–100]	65.0 (28.1) 60 [16–150]	0.612	64.9 (27.5) 60.0 [10–150]	57.1 (24.2) 50.0 [16.-100]	0.612
	< 70% of predicted	32 (67.3)	21 (65.6)	11 (55.0)	0.561	9 (60%)	23 (62.1%)	0.885	25 (61.0%)	7 (63.6%)	0.872

^a^*n* = 49, *** *p* < 0.001, ** *p* < 0.001, and * *p* < 0.05 compared to normal values

*FVC* Forced Vital Capacity: *FEV1* Forced Expiratory Volume during the first second: *FIVC* Forced Inspiratory Vital Capacity: *MIP* Maximal Inspiratory Pressure: *MEP* Maximal Expiratory Pressure: *6MWD* 6 Minute Walk Distance: *1MSTS* 1 min sit to stand test

Both mean MIP and MEP exceeded 80% of predicted values in both men and women. Women had a lower median MIP compared to men (− 18% vs. − 3%, *p* = 0.060). Patients who required prolonged mechanical ventilation (≥20 days) had higher MIP and MEP values compared with those ventilated for < 20 days, although these differences did not reach statistical significance. Moreover, no patient who required prolonged ventilation had an MIP below the LLN, while 14 patients (34%) ventilated for < 20 days had both MIP and MEP below the LLN (Table 2). Subgroup analysis revealed that 3 patients (27%) in the prolonged mechanical ventilation group had pulmonary disease (2 with asthma, 1 with COPD) before COVID-19. Two of these patients had sub-LLN levels of FEV and FEV1, but not FIVC.

For the whole group, the mean physical capacity measured by 6MWD was over 80% of predicted; however, 13 (26%) subjects showed results below the LLN [26]. The results of the 1MSTS were comparable to those during the 6MWD; however, most patients (73%) had a value below the LLN on the 1MSTS, and 9 (18%) desaturated during the test (SpO_2_ < 92%). Compared to patients without prior pulmonary disease, those with pulmonary disease before COVID-19 exhibited significantly inferior (18%) performance on the 6MWD (*p* = 0.028) and desaturated more often during the 1MSTS (*p* = 0.039). Over 90% of patients with previous pulmonary disease had an 1MSTS value below the LLN (i.e., below 73% of predicted). Patients who did versus did not require prolonged mechanical ventilation performed similarly on physical capacity tests, except that all who required a longer intubation time had a 1MSTS result below the LLN, which was a significantly a higher proportion (+ 35.2%) than in the comparison group.

Among all 52 patients, the mean thoracic expansion recorded was 63% (SD, 27%) of the predicted value, and 32 patients (67%) exhibited an expansion value below 70% of predicted. Thoracic expansion did not significantly differ according to gender, previous pulmonary disease, or duration of mechanical ventilation. There was a low correlation between thoracic expansion and measured lung volume: % predicted FVC (r_s_ = 0.360, *p* = 0.009), % predicted FEV1 (r_s_ = 0.270, *p* = 0.053), and % predicted FIVC (r_s_ = 0.301, *p* = 0.032).

**Fig. 2:**
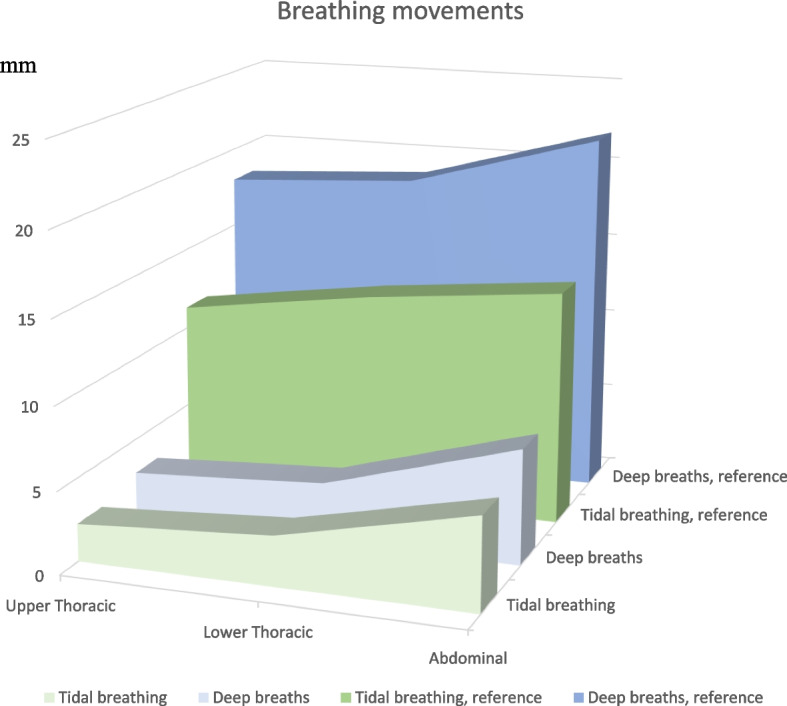
Breathing movements during tidal breathing and deep breaths in patients with remaining symptoms and their reference movements

Tables 3 and 4 present data regarding respiratory movements. The results were significantly different from the reference values in the upper thorax and abdomen during tidal volume breathing (*p* < 0.001), and at all three measuring points during deep breathing (*p* < 0.01). Of the 52 patients, 23 (47%) had a predominant breathing pattern in the upper thorax during tidal volume breathing, and 18 (40%) during deep breathing (Fig. 2). The respiratory movement measurements did not significantly differ between men and women, or according to whether patients experienced tightness around the chest or had previous diagnosed pulmonary disease. However, patients who had required prolonged mechanical ventilation exhibited significantly smaller breathing movements during both tidal volume and deep breathing (*p* < 0.05), except for a significantly larger abdominal movement during tidal breathing (*p* = 0.048). Patients who had not required ≥20 days of mechanical ventilation breathed more predominantly in the upper thorax.

**Table 3:** Respiratory movements in patients undergoing test with RMMI in the whole group and in men vs. women. Mean (SD), median [min-max]

			**All**, *n* = 49	**Men**, *n* = 32	**Women**, *n* = 17	*p*-**value**
**Tidal volume breathing**	**Upper thoracic**	% pred	77.9 (44.2)*** 66.6 [18.3–206.2]	75.0 (39.5)** 65.6 [23.6–109.4]	83.2 (52.9) 67.9 [18.3–206.2]	0.690
		< 70% of pred	28 (57.1%)	19 (59.4%)	9 (52.9%)	0.765
	**Lower thoracic**	% pred	85.4 (53.5) 71.5 [20.8–202.4]	82.0 (58.3) 62.4 [20.8–202.4]	91.8 (43.9) 85.6 [30.8–191.3]	0.193
		< 70% of pred	24 (49.0%)	18 (56.2%)	6 (35.3%)	0.232
	**Abdominal**	% pred	80.3 (70.5)*** 70.5 [16.0–255.1]	83.9 (48.1)** 77.6 [23.0–255.1]	73.4 (37.7)* 66.7 [16.0–135.5]	0.737
		< 70% of pred	23 (46.9%)	14 (43.8%)	9 (52.9%)	0.564
	**Upper thoracic/abd**	%	110.4 (59.8) 97.7 [31.4–358.3]	100.1 (31.4) 88.4 31.4–224.0]	129.8 (75.0) 102.0 [31.8–358.3]	0.544
		> 100% n (%)	23 (46.9%)	14 (43.8%)	9 (52.9%)	0.564
			**All,** ***n*** **= 45**	**Men,** ***n*** **= 29**	**Women,** ***n*** **= 16**	***p*-value**
**Deep breathing**	**Upper thoracic**	% pred	63.0 (56.4)*** 42.2 [4.2–180.6]	58 7 (55.7)** 55.7 [5.0–154.3]	70.7 (58.6) 83.4 [4.2–180.6]	0.776
		< 70% of pred	25 (56.8%)	18 (62.1%)	7 (43.7%)	0.192
	**Lower thoracic**	% pred	69.4 (63.9)** 23.1 [4.3–205.6]	65.0 (66.0)* 22.2 [4.7–205.6]	77.4 (61.3) 102.3 [4.3–159.8]	0.393
		< 70% of pred	24 (53.3%)	17 (58.6%)	7 (43.7%)	0.369
	**Abdominal**	% pred	64.2 (54.2)*** 29.2 [6.4–168.2]	61.9 (54.5)** 27.5 [6.4–168.2]	68.2 (55.2) 71.5 [7.8–148.6]	0.813
		< 70% of pred	24 (53.3%)	17 (58.6%)	7 (43.7%)	0.369
	**Upper thoracic/Abd**	%	108.7 (109.3) 82.1 [25.7–703.9]	106.3 (124.6) 75.0 [25.7–703.9]	113.1 (77.5) 89.0]27.8–314.6]	0.801
		> 100%, n (%)	18 (40.0%)	12 (41.4%)	6 (35.3%)	0.999

*** *p* < 0.001, **p < 0.01, *p < 0.05 vs. reference values

**Table 4:** Results of respiratory movements measured by RMMI related to previous pulmonary disease and need of prolonged mechanical ventilation. Mean (SD), median [min-max]

			**Previous** **pulmonary** **disease**			**Mechanical ventilation**		
			Yes *n* = 14	No *n* = 35	*p*-value	< 20 days *n* = 38	≥ 20 days *n* = 11	*p*-value
**Tidal volume breathing**	**Upper thoracic**	% pred	76.6 (48.6) 59.5 (23.6–206.2]	78.4 (43.1) 68.5 [18.3–204.1]	0.677	84.2 (47.0) 71.0 [18.3–206.2]	56.0 (23.1) 53.5 [28.7–105.3)	**0.045**
		< 70% of pred	9 (64.3%)	13 (37.1%)	0.750	19 (46.3%)	9 (81.8%)	**0.087**
	**Lower thoracic**	% pred	66.9 (45.0) 62.3 [20.8–191.3]	92.8 (55.3) 74.2 [21.2–202.4]	0.132	91.3 (49.7) 77.4 [28.1–199.3]	65.2 (63.4) 44.1 [20.8–202.4]	**0.023**
		< 70% of pred	8 (57.1%)	16 (45.7%)	0.538	15 (36.5%)	9 (81.8%)	**0.018**
	**Abdominal**	% pred	70.5 (36.7) 80.4 [16.0–128.5]	84.2 (47.4) 70.4 [21.8–255.19	0.582	78.4 (39.6) 77.6 [16.0–220.3]	86.9 (60.7) 65.5 [35.3–255.1]	**0.048**
		< 70% of pred	6 (42.9%)	17 (48.6%)	0.761	16 (39.0%)	7 (63.6%)	0.306
	**Upper thoracic/abd**	%	130.8 (84.7) 95.1 [38.7–358.3]	102.2 (45.4) 97.7 [31.4–207.9]	0.493	121.1 (62.9) 106.7 [31.4–358.3]	73.1 (23.7) 71.2 [41.3–113.0]	**0.006**
		> 100% n (%)	6 (42.9%)	16 (45.7%)	0.761	21 (51.2%)	2 (18.2%)	**0.042**
			Yes *n* = 11	No *n* = 31	*p*-value	< 20 days *n* = 34	≥20 days *n* = 11	*p*-value
**Deep breathing**	**Upper thoracic**	% pred	59.4 (62.4) 17.0 [5.5–180.6]	64.6 (54.5) 56.6 [4.2–154.3]	0.797	74.0.6990 (58.4) 83.4 [4.2–180.6]	28.9 (32.1) 17.3 [4.2–104.3]	**0.039**
		< 70% of pred	8 (72.7%)	17 (54.8%)	0.885	16 (47.1%)	9 (81.8%)	0.079
	**Lower thoracic**	% pred	63.2 (64.5) 21.3 [4.3–164.5]	72.3 (64.5) 65.6 [4.7–205.6]	0.500	79.7 (61.4) 94.0 [4.7–202.4]	37.6 (63.9) 8.3 [4.3–205.6]	**0.024**
		< 70% of pred	8 (72.7%)	16 (51.6%)	0.759	15 (44.1%)	9 (81.8%)	**0.040**
	**Abdominal**	% pred	50.0 (51.0) 21.6 [6.4–165.0]	70.6 (55.2) 71.0 [8.4–168.2]	0.230	72.4 (56.2) 76.8 [6.4–168.2]	38.8 (39.8) 21.3 [11.2–127.8]	0.302
		< 70% of pred	9 (81.8%)	15 (48.4%)	0.356	15 (44.1%)	9 (81.8%)	**0.040**
	**Upper thoracic/Abd**	%	149.0 (173.6) 96.9 [29.3–703.9]	90.5 (58.0) 82.1 [25.7–314.6]	0.377	120.0 (120.9) 87.2 [25.7–703.9]	73.7 (50.3) 56.3 [29.3–202.3]	0.089
		> 100%,n (%)	7 (63.6%)	11 (35.5%)	0.512	15 (44.1%)	3 (27.3%)	0.482

*** *p* < 0.001, **p < 0.01, *p < 0.05 vs. reference values §

Table 5 presents the measured abnormal functions and the various combinations. The participants had a median of two abnormal functions, with a range from none to all six included in the analysis. We identified 27 combinations of abnormal functions among the 52 patients and did not identify any typical pattern for this category of patients.

**Table 5:**
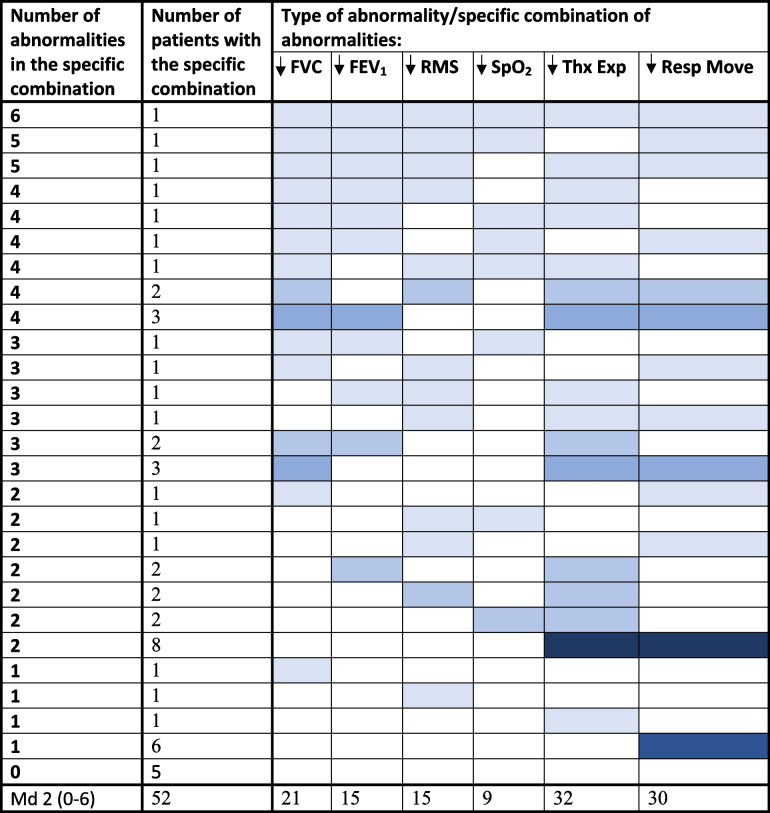
A presentation of the abnormal functions and the various combinations. The darker colour the more common abnormality/combination of abnormalities, no colour represents no abnormality

*FVC* Forced Vital Capacity: *FEV*_*1*_ Forced Expiratory Volume during the first second: *RMS* Respiratory Muscle Strength: *Thx Exp* Thoracic Expansion: *Resp Move* Respiratory Movement

## Discussion

In this study, we aimed to describe patients’ various remaining respiratory symptoms at 4 months after discharge from hospitalisation due to COVID-19, and to analyse whether these symptoms were influenced by sex, previous pulmonary disease, or prolonged mechanical ventilation. These results showed that at 4 months post-hospital discharge:

- Over 40% of patients had FVC below the LLN, and nearly 30% had FEV1 below the LLN.
- Nearly 30% of patients had MIP below the LLN, and women appeared more severely affected than men. MEP appeared to be less affected than MIP.
- The 1MSTS performance was below the LLN in > 70% of patients.
- Respiratory movements were below normality in over half of the participants, independent of sex. Thoracic expansion was reduced in 62% of the participants.

Our present results also revealed that patients with prior pulmonary disease had lower recorded 6MWD results, and > 90% had 1MSTS performance below the LLN. Furthermore, compared to those without previous pulmonary disease, patients with prior pulmonary disease were more likely to have SpO_2_ < 92% during the 1MSTS. Additionally, patients who required prolonged mechanical ventilation exhibited significantly poorer 6MWD performance, and all these patients had 1MSTS results below the LLN at 4 months post-discharge. However, lung function did not significantly differ between these groups.

Decreased lung function has been reported among patients after discharge from hospitalisation due to COVID-19 [1, 3, 33, 34]. The present findings confirmed this pattern, with patients showing significantly lower FVC and FEV1 (both *p* < 0.001). These results showed only minor and non-significant impacts of sex, presence of pulmonary disease before COVID-19, and need of prolonged mechanical ventilation. The FVC and FEV1 values were lower than those reported in earlier trials, possibly because our current study cohort included only patients with remaining respiratory symptoms [33, 34]. The lower values may also be due to residual effects of ventilator use (which could not be confirmed), or because the infection caused greater trauma to the lung parenchyma in these patients. Notably, although FVC and FEV1 were affected, FIVC was not significantly different from the reference values. This indicates that some of the subjects may have had bronchial obstruction at the time of examination, and that further spirometry evaluation would have been valuable.

As in previous publications, our patients exhibited decreased physical capacity measured as 6MWD at several months after discharge (82 ± 24% of predicted values) [33]. Furthermore, 18% of patients desaturated during this test. The patients with prior pulmonary disease or who needed prolonged mechanical ventilation, had significantly shorter walking distance during the 6MWD (*p* < 0.05). Compared to our present total cohort, our previous study of non-hospitalised patients exhibited slightly worse physical capacity (73 ± 24%), although a lower proportion (12%) desaturated [16]. These findings are likely at least partly due to the current subjects’ immobilisation while hospitalised and need for intensive care (60%). On the other hand, non-hospitalised patients desaturated during testing without that impact of hospitalization and low lung volumes. In a study conducted at Jin Yin-tan Hospital (Wuhan, China) during 2020, 1733 patients were examined at 6 months after hospital discharge due to COVID-19 [33], and the 6MWD was conducted among a battery of other tests. The physical capacity was below the LLN in 24% of patients who had been hospitalised but did not require supplemental oxygen, 22% of patients who were hospitalised and required supplemental oxygen, and 29% of patients who were hospitalised and needed invasive or non-invasive ventilation. The corresponding proportions of patients with pulmonary diffusion capacity below the LLN in these three groups were 22, 29, and 56%. Dyspnoea was reported in 26% of patients, and FEV_1_ and FVC values were < 80% of predicted in less than 23 and 15% of the whole study cohort, respectively [33]. These findings are in line with other reports showing that traditional lung function tests measuring volumes and flows do not capture the patients’ subjective experience of breathing discomfort, nor explain the decreased diffusion capacity or dyspnoea rating [3].

As earlier trials have not managed to fully capture the problems suffered by post-COVID-19 patients, in the present study, we included more functional measures, such as respiratory muscle strength (MIP and MEP), and the respiratory movement during tidal volume breathing and deep breathing. In our previous study among non-hospitalised patients, 64% exhibited reduced MIP, and 17% exhibited reduced MEP [16]. The corresponding rates in our current study were 27 and 8%. Surprisingly, compared to patients who did not require prolonged mechanical ventilation, those who had needed prolonged mechanical ventilation less commonly had MIP levels below the LLN. One hypothesis is that these patients involuntarily practiced expiratory muscle training while coughing during and after their hospitalisation. Regarding breathing patterns, the entire patient cohort exhibited decreased movements in all three positions (upper thoracic, lower thoracic, and abdominal) during both tidal volume breathing and deep breathing. Reduced breathing movement in the thorax was more predominant among patients who needed prolonged mechanical ventilation, compared to those who did not. In patients with prolonged mechanical ventilation, the breathing pattern was primarily abdominal, in contrast to the other group who exhibited larger breathing movement in the upper thorax. The pattern involving mostly thoracic movement was also observed in our previous study of non-hospitalised patients. In that study, breathing predominantly in the upper part of thorax was observed in one out of four patients during tidal volume breathing, and half of the participants during deep breathing [16]. It is unclear what caused these between-group differences in respiratory movement, especially during tidal volume breathing, but possibly explanations include chronic autonomic dysfunction caused by remaining viruses, persistent inflammation, affected sensing systems, or circulating antibodies resulting in diminished diaphragm function [6].

Since most traditional lung function and other commonly used tests are often within normal values, patients with remaining respiratory dysfunction often feel that they are not believed and express feelings of embarrassment when they contact healthcare services [3]. It may be an important complement to standard care for these patients to be assessed by a multi-professional team, including a physiotherapist specialised in pulmonary rehabilitation. During such a session, the patients’ symptoms may be further understood and confirmed. Measurements beyond the traditional battery may capture what the patients are experiencing and facilitate an individualised and improved functional rehabilitation. For this purpose, we recommend the additional use of measurements of breathing pattern and breathing muscle strength.

The detailed assessment of both abnormal functions and subjective symptoms could facilitate the effects of rehabilitation. Physiotherapists who are experienced in respiratory medicine may detect abnormal breathing patterns when examining patients. To individualise the rehabilitation program for patients suffering from post-COVID-19, it is crucial to detect specific abnormalities not only in the acute phase but also repeatedly during the rehabilitation process [3, 7–10]. The test results are also critical for determining which patients need treatment, and how to tailor and optimise the treatment strategy. A large variety of treatments can be used to restore breathing function, such as respiratory muscle training and retraining of a normal breathing pattern. Although the level of evidence for these treatments is currently low, several trials are ongoing, which will hopefully provide more knowledge within the coming years.

Our study has both strengths and limitations. One strength is the thorough, standardised, and expanded examination by specialised physiotherapists, which even captured the variation among patients’ subjective symptoms. The patients were included from all of the different standard levels of public hospitals—from university to local county hospitals—in a region of Sweden. This study was based on a cohort of consecutive patients, generating a group that was heterogenous in several aspects, such as age, socioeconomic background, and educational level. However, due to the exclusion criteria, the group was selected to a certain extent. For instance, this sub-cohort did not contain any patients who needed supplementary oxygen at discharge, or with known lung parenchyma fibrosis. Addition, this study included only a few patients from the first wave of the pandemic. Another limitation is the small sample size, which must be kept in mind when interpreting the results regarding differences between the subgroups.

Overall, the presently available data suggest that it is highly important to follow-up individual patients with multifactorial tests. The results can support an individualised and continuously optimised rehabilitation regimen, as the symptoms vary among patients and over time. This knowledge may encourage caregivers to focus on patients with remaining respiratory symptoms, and to adequately respond to persistent symptoms by engaging a multidisciplinary team earlier in the rehabilitation process.

In conclusion, this study showed that patients with remaining respiratory symptoms at 4 months after discharge from hospitalization due to COVID-19 may suffer from various abnormal breathing functions, and dysfunctional breathing that is normally not detected by traditional measurements. Therefore, we recommend the regular use of a more multidimensional protocol for measuring breathing movements, thoracic expansion, and respiratory muscle strength, in addition to traditional measurements. This will help assess the symptoms that patients experience and enable the prescription of optimal treatment interventions and rehabilitation programs.

## Data Availability

The datasets used and analysed during the current study are available from the corresponding author on reasonable request.
